# Effectiveness of omalizumab in patients with severe allergic asthma with and without chronic rhinosinusitis with nasal polyps: a PROXIMA study post hoc analysis

**DOI:** 10.1186/s13601-020-00330-1

**Published:** 2020-06-26

**Authors:** Enrico Heffler, Fabiana Saccheri, Marta Bartezaghi, Giorgio Walter Canonica

**Affiliations:** 1 Personalized Medicine, Asthma and Allergy, Humanitas Clinical and Research Center IRCCS, Via Alessandro Manzoni 56, 20089 Rozzano, MI Italy; 2 Department of Biomedical Sciences, Humanitas University, Via Rita Levi Montalcini 4, 20090 Pieve Emanuele, Mi Italy; 3 Medical & Scientific Department, Novartis Farma SpA, Origgio, Italy

**Keywords:** Biologics, Exacerbations, Nasal Polyps, Observational, Omalizumab, Severe asthma

## Abstract

**Background:**

A significant proportion of patients with severe asthma may also suffer from nasal polyposis, which is commonly defined as chronic rhinosinusitis with nasal polyps (CRSwNP), the presence of which may adversely affect asthma treatment outcomes. The biologic agent omalizumab is effective as add-on therapy in patients with severe allergic asthma. The aim of this post hoc analysis of the PROXIMA study was to compare the efficacy of omalizumab between patients with severe allergic asthma, with and without comorbid CRSwNP.

**Methods:**

PROXIMA was a prospective observational 2-part study conducted in Italy in adult patients with severe allergic asthma, where, in the second part, patients eligible for add-on omalizumab initiated treatment for 12 months. Patient baseline data such as comorbidities and history of exacerbations were collected. Outcomes were asthma control (Asthma Control Questionnaire [ACQ]), lung function (forced expiratory volume in 1 s [FEV_1_]) and exacerbation rate. The post hoc analysis compared these outcomes between the cohort with comorbid CRSwNP and the cohort without CRSwNP.

**Results:**

Of 123 patients included in this analysis, 17 (13.8%) were in the CRSwNP cohort. There was no significant difference between cohorts in baseline clinical characteristics or in change from baseline at 12 months in ACQ values,  % of predicted FEV_1_ or annual asthma exacerbation rate, although results were numerically in favor of the CRSwNP cohort versus the non-CRSwNP cohort. The proportion of patients who achieved an improvement in all three outcomes was numerically greater in the CRSwNP cohort (35.7% vs 23.0%).

**Conclusions:**

In an observational real-world setting, add-on omalizumab for severe allergic asthma was effective in improving asthma control, lung function and in reducing exacerbations, including in those patients with CRSwNP.

## Background

Asthma is one of the most common non communicable diseases and is prevalent worldwide, affecting more than 300 million individuals [1]. Key characteristics of asthma include respiratory symptoms (e.g. dyspnea, wheeze), caused by chronic airway inflammation, that vary in severity over time and expiratory airflow limitation that is also variable [1].

There is now growing evidence for different phenotypes within severe asthma, defined by the characteristic clinical manifestations, pathological and physiological mechanisms and biomarkers [2]. Starting from the classification of type 2 (T2) and not type 2 (not T2), asthma can be characterized as allergic or non-allergic within the phenotype/endotype T2, in which the eosinophilic component can be more or less prevalent [2].

After the optimization of the therapy, the verification of the adherence of patients, and the treatment of possible comorbidities, a diagnosis of severe asthma could be confirmed if the patient is still uncontrolled according to the international guidelines [1, 3].

Disease control can be achieved in many patients with asthma through the chronic use of anti-inflammatory agents—usually inhaled corticosteroids [ICS]—frequently in combination with bronchodilators such as long-acting β_2_-agonists. A small proportion of patients however continue to experience symptoms and frequent asthma exacerbations despite treatment with high-dose ICS in combination with other controller drugs and/or chronic use of oral corticosteroids (OCS): these patients are considered to have severe asthma [3]. Novel biologic drugs have been developed to treat severe asthma by targeting specific immunologic mechanisms [4], complying therefore with the concepts of personalized and precision medicine [5].

First approved more than 15 years ago, omalizumab was the first biologic agent available for the treatment of severe allergic asthma [6]. Omalizumab is a humanized, recombinant, anti-human immunoglobulin E (IgE) antibody derived from murine monoclonal antibodies [7], with proven efficacy and effectiveness for adult and pediatric patients with severe allergic asthma [8–10]. Omalizumab treatment is associated with significant reductions in asthma exacerbation rates and OCS use, and improvements in asthma-related quality of life [10–13]. Based on these results, omalizumab was first approved in 2003 in the US for moderate-to severe allergic asthma and then later in 2005 in Europe for severe allergic asthma. Omalizumab as add-on treatment for severe allergic asthma was shown to be cost-effective, decreasing both direct and indirect costs related to the disease [14].

Disease control may be influenced by certain comorbidities in patients with severe asthma; these comborbidities may also lead to reduced efficacy of both standard and biologic therapy, and may impact on asthma exacerbations [15–17]. Among severe asthma comorbidities, one of the most frequent and relevant is nasal polyposis, commonly defined as chronic rhinosinusitis with nasal polyps (CRSwNP) [18], which affects at least 30% of patients with severe asthma [16, 19–21]. CRSwNP shares immunologic mechanisms with severe asthma [22], particularly with those endotypes characterized by higher expression of type-2 biomarkers [23, 24] such as allergic and non-allergic but eosinophilic asthma [25].

In addition, a subgroup of patients with CRSwNP and asthma may experience worsening of the two diseases’ symptoms, and asthma exacerbations, following treatment with non-steroidal anti-inflammatory drugs (NSAIDs), otherwise known as NSAID-Exacerbated Respiratory Disease (N-ERD) [26]. N-ERD is a clinical syndrome that typically includes hypersensitivity to aspirin and other NSAIDs, CRSwNP, and asthma (even if some patients may have only asthma or only CRSwNP associated to NSAIDs hypersensitivity) and it is often associated with the most severe outcomes of both asthma and CRSwNP [27]. Regardless of the presence or absence of N-ERD, patients with severe asthma plus CRSwNP tend to have worse asthma control [28], more severe asthma [29], a reduced likelihood of asthma remission [30] and higher airway inflammation [31, 32]. Moreover, serum total IgE concentration has been found to be a good biomarker for eosinophilic nasal polyps and comorbid asthma [33, 34].

The aim of this post hoc analysis of the Patient-Reported Outcomes and Xolair^®^ In the Management of Asthma (PROXIMA) study [9, 35] was to evaluate the effectiveness of omalizumab in severe allergic asthmatic patients stratified by the presence of concomitant CRSwNP.

## Methods

### Study design and patients

The study design has been reported in detail previously [35]. Briefly, PROXIMA was an observational study composed of a cross-sectional phase followed by a prospective 12-month longitudinal observational phase conducted between December 2013 and June 2016 at 25 hospital and academic centers in Italy [35]. Study objectives were to ascertain the prevalence of perennial allergic asthma in the Italian population and determine the proportion of omalizumab recipients who achieved and sustained asthma control.

Main inclusion criteria in the cross-sectional phase were patients with severe allergic asthma aged ≥ 18 years currently receiving Global Initiative for Asthma step 4 therapy requiring a step up in therapy [35]. Of these patients, those who initiated treatment with subcutaneous omalizumab as per clinician judgment and according to the Italian approved indications and reimbursement criteria (severe asthma, with a FEV1 < 80% predicted, sensitized to perennial allergens and with a serum IgE of 30–1500 U/mL, provided that the expected dosage based on body weight and serum IgE levels does not exceed 600 mg every 2 weeks) entered the longitudinal observational follow-up phase, with follow-up visits at 6 and 12 months [35].

As previously described, the study was conducted in accordance with the ethical principles laid down in the Declaration of Helsinki, the Italian Medicines Agency (AIFA) Guidelines for classification and management of observational studies on drugs, and the Strengthening the Reporting of Observational Studies in Epidemiology statement [36–38]. All patients provided informed consent before participating in the study.

Patient data collected at baseline of the cross-sectional phase included the presence of major asthma comorbidities, and assessments conducted at baseline and study end included asthma control (Asthma Control Questionnaire [ACQ] [39]) and lung function (forced expiratory volume in one second [FEV_1_]). The number of asthma exacerbations in the 12 months before and after the onset of omalizumab treatment were also analyzed. Since the evaluation of comorbidities was not an endpoint of the primary study, CRSwNP-specific outcomes (such as symptoms, endoscopic NP score, CRS exacerbations, operations) were not monitored during the study.

### Post hoc analysis objective

The main objective of this post hoc analysis of the PROXIMA study was to compare the effect of 12 months’ omalizumab treatment on the change in ACQ scores, FEV_1_ and the number of asthma exacerbations (defined as deterioration of asthma symptoms that required the use of ‘rescue’ treatment such as systemic corticosteroids or an increase in the patient’s maintenance systemic corticosteroids for at least 3 days, or hospitalization or emergency department admission) in patients with severe allergic asthma with CRSwNP compared with those without CRSwNP at baseline. Thus patients were stratified by the presence or absence of CRSwNP [18] in the medical history section of their case report form.

### Statistical analysis

Demographic and baseline characteristics were summarized using descriptive statistics such as mean, standard deviation (SD), median, interquartile range, minimum and maximum for continuous variables and absolute and relative frequencies for categorical variables. Non-parametric Wilcoxon test or Analysis of Covariance (ANCOVA) on ranks was used to analyze differences between cohorts in age, body mass index, time from asthma diagnosis, omalizumab dose, FEV1 and number of exacerbations. Parametric test, as *t* test or ANCOVA, was used for detecting difference between cohorts in ACQ. A simple Chi square test was used for detecting difference in proportion (e.g. gender).

## Results

### Patients

Of the 365 patients included in the cross-sectional phase of the PROXIMA study, 123 entered the longitudinal phase [9] and were included in the present analysis. The distribution of main asthma comorbidities at baseline is reported in Table 1. Twenty-four patients (19.5%) had only one comorbidity, 30 (24.4%) had two comorbidities and the remaining 23 (18.7%) reported more than two comorbidities.

**Table 1:** Distribution of main asthma comorbidities in the PROXIMA study population

Comorbidity	N = 123
Number of patients with at least one comorbidity	77 (62.6%)
Allergic bronchopulmonary aspergillosis	2 (1.6%)
Atopic dermatitis	3 (2.4%)
Bronchiectasis	3 (2.4%)
Cardiovascular disease	26 (21.1%)
Chronic obstructive pulmonary disease	4 (3.3%)
Chronic rhinitis	17 (13.8%)
Chronic rhinosinusitis without nasal polyps (CRSsNP)	22 (17.9%)
Chronic rhinosinusitis with nasal polyps (CRSwNP)	17 (13.8%)
Chronic spontaneous urticaria	1 (0.8%)
Chronic/recurrent respiratory infections	2 (1.6%)
Gastroesophageal reflux	16 (13.0%)
Hormonal disturbances	16 (13.0%)
Obesity	5 (4.1%)
Obstructive sleep apnea/sleep-disordered breathing	3 (2.4%)
Psychologic disease (anxiety, depression, behavioral disorders)	6 (4.9%)
Other	19 (15.4%)

CRSwNP was present in 17 (13.8%) patients (10 female, mean (SD) age 51.6 ± 12.5 years).

Their clinical and functional features did not differ from the cohort without CRSwNP (Table 2): in particular, baseline mean (SD) ACQ scores (2.87 ± 1.60 vs 2.98 ± 1.02, p = 0.7065), FEV_1_ (1.74 ± 0.80 L vs 1.70 ± 0.72 L, p = 0.7347) and annual asthma exacerbation rate (5.13 ± 4.13 vs 4.54 ± 4.08, p = 0.4131) were similar in the two groups.

**Table 2:** Baseline clinical and demographic patient characteristics

	Patients with CRSwNP (n = 17)	Patients without CRSwNP (n = 106)	*p* value
Age (years)	51.6 ± 12.5	52.9 ± 13.7	0.626
Female (%)	58.8	62.3	0.786
BMI (kg/m^2^)	24.8 ± 4.1	26.4 ± 4.8	0.155
Time from asthma diagnosis (years)	12.9 ± 9.9	18.6 ± 14.0	0.161
FEV_1_ (l)	1.74 ± 0.80	1.70 ± 0.72	0.735
FEV_1_ % predicted, median (Q1–Q3)	61.01 (48.53–70.58)	54.18 (45.65–69.23)	0.690
ACQ	2.87 ± 1.60	2.98 ± 1.02	0.706
No. of asthma exacerbations in the previous 12 months	5.13 ± 4.13	4.54 ± 4.08	0.413
Monthly dose of omalizumab, mg	553.13 ± 303.57	521.23 ± 331.68	0.5233

Data are mean ± SD unless otherwise stated

*ACQ* Asthma Control Questionnaire, *BMI* body mass index, *FEV*_*1*_ forced expiratory volume in 1 s, *CRS*w*NP* chronic rhinosinusitis with nasal polyps, *SD* standard deviation

### Efficacy

**Fig. 1:**
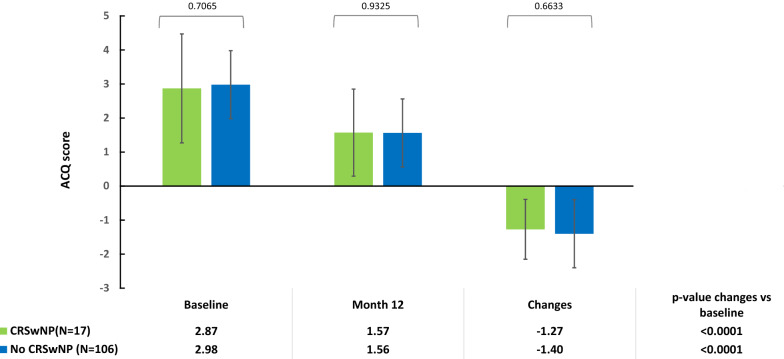
Mean (standard deviation) Asthma Control Questionnaire scores at baseline and 12 months after omalizumab treatment, and the change from baseline in ACQ score, in patients with severe allergic asthma, with chronic rhinosinusitis with nasal polyps (CRSwNP) or without CRSwNP (No CRSwNP). The p-values within cohorts were calculated using a signed rank test and p-values for comparisons between cohorts were calculated using an ANCOVA model on ranks

**Fig. 2:**
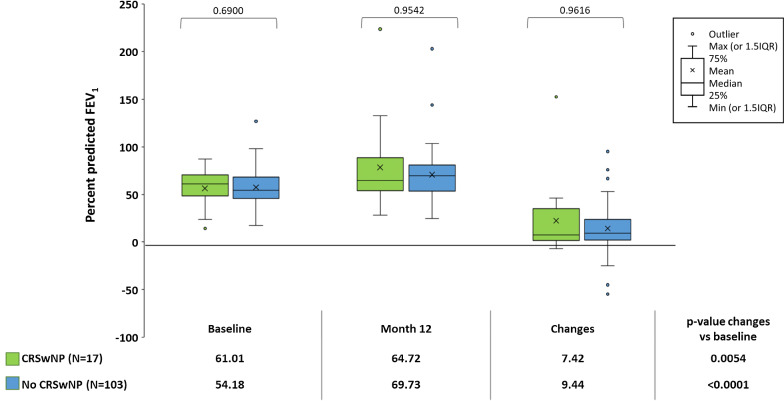
Lung function assessed via percent predicted forced expiratory volume in 1 s (FEV_1_) at baseline and 12 months’ after omalizumab treatment, and change from baseline in percent predicted FEV_1_, in patients with severe allergic asthma, with chronic rhinosinusitis with nasal polyps (CRSwNP) or without CRSwNP (No CRSwNP). The p-values within cohorts were calculated using a signed rank test and p-values for comparisons between cohorts were calculated using an ANCOVA model on ranks

**Fig. 3:**
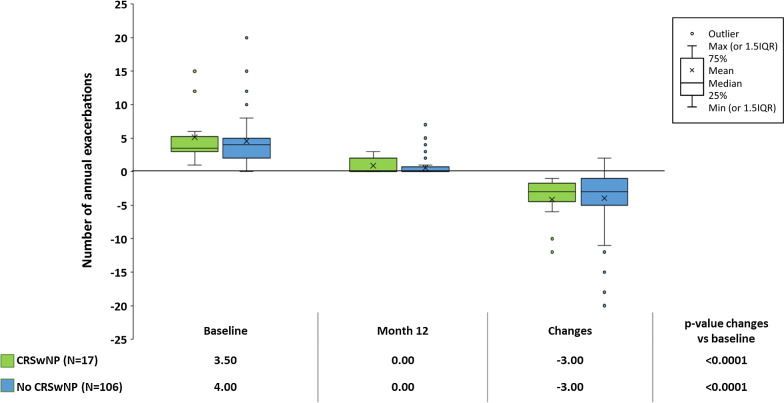
The median number of annual exacerbations in the year prior to initiating omalizumab treatment (baseline) and during 12 months’ treatment with omalizumab, and change from baseline, in patients with with severe allergic asthma with chronic rhinosinusitis with nasal polyps (CRSwNP) or without CRSwNP (No CRSwNP). The p-values within cohorts were calculated using a signed rank test and p-values for comparisons between cohorts were calculated using an ANCOVA model on ranks

At 12 months both patients with and without CRSwNP, with no difference between cohorts, achieved an improvement from baseline in ACQ values (mean change: – 1.27 ± 0.88, p < 0.0001 vs –1.40 ± 1.19, p < 0.0001 respectively; p-value between the two groups: 0.6633) (Fig. 1), in  % of predicted FEV_1_ (median change: +7.42 (0.44–35.00) liters, p = 0.0054 vs +9.44 (1.85–23.89), p < 0.0001; p-value between the two groups: 0.9616) (Fig. 2) and in annual asthma exacerbation rate (median change: – 3.00 (– 5.00 to – 1.50), p < 0.0001 vs –3.00 (–5.00 to –1.00), p < 0.0001; p-value between the two groups: 0.2517) (Fig. 3). Moreover, we also obtained similar results in patients with chronic sinusitis/rhinosinusitis, patients with rhinitis and patients with only asthma (Additional file 1: Fig. S1, Additional file 2: Fig. S2 and Additional file 3: Fig. S3).

A total of 40.0% of patients with CRSwNP compared with 28.1% of those without CRSwNP achieved good asthma control defined as ACQ < 1 at the end of the 12-month period of treatment (p = 0.3509). Moreover, patients with CRSwNP also had a greater numerical improvement in FEV_1_ compared with those without CRSwNP. Similarly, all the patients included in the CRSwNP cohort showed a reduction in the number of exacerbations after 12 months of omalizumab treatment, compared with the 85.4% of patients in the cohort without CRSwNP (p = 0.1025). The proportion of patients who achieved an improvement in all three of the outcomes (ACQ, FEV_1_ and asthma exacerbations) was 35.7% of the cohort with CRSwNP versus 23.0% of those without CRSwNP (p = 0.3127), and all patients with CRSwNP achieved an improvement in at least one of the three outcomes while 6.8% of those without CRSwNP did not respond to any outcome (p = 0.3166).

## Discussion

In this post hoc analysis of the real-world PROXIMA study [9, 35], we evaluated the effectiveness of omalizumab in patients with severe allergic asthma in two cohorts stratified by the presence/absence of comorbid CRSwNP. The presence of CRSwNP did not negatively influence the response to omalizumab treatment in terms of improvement in asthma control and lung function or in reduction of annual asthma exacerbation rate.

Our findings are in line with those recently reported by Tiotiu et al. in a retrospective, multicenter study that included patients with severe allergic asthma and CRSwNP treated with omalizumab for 6 months [40]. They observed an improvement in all lower airways clinical outcomes in this particular subgroup of patients with severe asthma. In another retrospective case–control study in 259 patients with severe allergic asthma treated with omalizumab, Clavenna et al. observed a significant improvement in lung function only in patients with comorbid chronic rhinosinusitis (they did not report the presence or absence of nasal polyps), who comprised 73% of the patient population [41]. Moreover, a recent Italian prospective observational study showed that CRSwNP was more common among patients achieving an improvement in lung function with normalization of FEV_1_ values than in those who did not obtain reversibility of airway obstruction and, together with rhinitis, the presence of CRSwNP was the best predictor of airway obstruction reversibility after omalizumab treatment [42]. Therefore, the latter study demonstrated that the long-term efficacy of omalizumab in severe allergic asthmatic patients is greater in those with CRSwNP as a comorbidity. In the present study, the effect of omalizumab on lung function was greater in patients with CRSwNP and, anyway, quite large (> 400 mL FEV1 improvement) and higher than the proposed Minimal Clinically Important Difference (MCID) for this outcome (> 15% from baseline values [43]) in all treated patients; this can be at least partially explained by the known positive effect of regular and frequent follow-ups (as for omalizumab administration) on adherence to asthma medications [44]. Moreover reported changes in FEV1 after 12 months of treatment are in line with another publication, which reported a significant improvement in FEV1 from 1636 ± 628.4 mL at baseline to 2000 ± 679.7 mL (p < 0.05) after 1 year of omalizumab treatment [45]. It is noteworthy that the improvement in ACQ and exacerbation rate in our study also surpassed their MCIDs [39, 46].

The effect of omalizumab in patients with severe allergic asthma associated with CRSwNP may suggest a possible concomitant effect of the drug on nasal polyps outcomes, as previously shown [40, 47]. The efficacy of omalizumab in patients with CRSwNP, with or without asthma, was recently confirmed in two phase III studies (POLYP 1 [48] and POLYP 2 [49]), where omalizumab met both co-primary endpoints and multiple key secondary endpoints, showing significant improvements in health-related quality of life, smell, congestion, endoscopic nasal polyp size, post-nasal drip, and runny nose [50].

All these data confirm that severe asthma and comorbid CRSwNP represent a distinct phenotype that may benefit from the use of biologic agents, including anti-IgE strategies, as already suggested by Rivero and Liang in their systematic review and meta-analysis on anti-IgE and anti-interleukin 5 (IL5) treatment for CRSwNP [51]. They showed that patients who have the greatest benefit from omalizumab in terms of improvement of CRSwNP outcomes are those patients with concomitant asthma [51].

Recently the European Forum for Research and Education in Allergy and Airway Diseases consortium published a position paper on the use of biologics in the treatment of CRSwNP, proposing that biologics be positioned into the care pathways for CRSwNP patients with or without asthma, and proposing criteria for the identification of patients who may benefit from treatment with biologics [52].

Our study is not without limitations, the main one being the small number of patients with comorbid CRSwNP; this is probably due to the fact that the primary study was not designed to evaluate comorbidities of severe allergic asthma, and this may have led to under diagnosis of CRS in a proportion of patients. However, the study showed that omalizumab is effective in severe asthma groups, both with or without CRSwNP. Another limitation is that the assessment of comorbidities was not predefined in the original protocol, so we were reliant on the investigators’ reports of the patients’ clinical history and available clinical data. Finally, we did not collect data on CRSwNP-specific outcomes because this was a post hoc analysis, and our analysis was confined to the data collected in the primary study.

## Conclusions

In conclusion, despite the relatively small number of patients included in our study, our data have shown that omalizumab has a beneficial effect on ACQ score, FEV_1_ values and exacerbation rate in severe allergic asthma patients also when a relevant comorbidity that may seriously impact on asthma severity itself, like CRSwNP, is present [15]. These findings are relevant as they reveal a real-life aspect of omalizumab treatment for severe allergic asthma that may have been underestimated or not taken in consideration during randomized clinical trials. Asthma and CRSwNP represent two aspects of a peculiar disease phenotype and may both represent treatable traits, particularly when considering biologic therapies [53, 54]. Further studies are needed to confirm our results; specifically, analysis of real-world ‘big data’ obtained from clinical registries may provide relevant information on the effectiveness of omalizumab in patients with severe allergic asthma and comorbid CRSwNP. Such studies also have the potential to investigate the impact of omalizumab on CRSwNP outcomes.

## Supplementary information

**Additional file 1: Figure S1.** Efficacy comparison of outcome parameters at baseline and 12 months after omalizumab treatment in patients with chronic rhinosinusitis with nasal polyps (CRSwNP) compared with chronic sinusitis/rhinosinusutis without (CRSsNP). (A) Mean Asthma Control Questionnaire scores, and the change from baseline in ACQ score; (B) Median percent predicted forced expiratory volume in 1 s (FEV_1_), and change from baseline in percent predicted FEV_1_; (C) Median number of annual exacerbations in the year prior to initiating omalizumab treatment (baseline) and during 12 months’ treatment with omalizumab, and change from baseline. The p-values within cohorts were calculated using a signed rank test and p-values for comparisons between cohorts were calculated using an ANCOVA model on ranks.

**Additional file 2: Figure S2.** Efficacy comparison of outcome parameters at baseline and 12 months after omalizumab treatment in patients with chronic rhinosinusitis with nasal polyps (CRSwNP) compared with rhinitis. (A) Mean Asthma Control Questionnaire scores, and the change from baseline in ACQ score; (B) Median percent predicted forced expiratory volume in 1 s (FEV_1_), and change from baseline in percent predicted FEV_1_; (C) Median number of annual exacerbations in the year prior to initiating omalizumab treatment (baseline) and during 12 months’ treatment with omalizumab, and change from baseline. The p-values within cohorts were calculated using a signed rank test and p-values for comparisons between cohorts were calculated using an ANCOVA model on ranks.

**Additional file 3: Figure S3.** Efficacy comparison of outcome parameters at baseline and 12 months after omalizumab treatment in patients with chronic rhinosinusitis with nasal polyps (CRSwNP) compared with severe asthma (no chronic rhinosinusitis or nasal polyps). (A) Mean Asthma Control Questionnaire scores, and the change from baseline in ACQ score; (B) Median percent predicted forced expiratory volume in 1 s (FEV_1_), and change from baseline in percent predicted FEV_1_; (C) Median number of annual exacerbations in the year prior to initiating omalizumab treatment (baseline) and during 12 months’ treatment with omalizumab, and change from baseline. The p-values within cohorts were calculated using a signed rank test and p-values for comparisons between cohorts were calculated using an ANCOVA model on ranks.

## Data Availability

The datasets generated and/or analyzed during the current study are not available for public disclosure.

## Abbreviations

ACQ: Asthma Control Questionnaire
FEV_1_: Forced expiratory volume in 1 s
IgE: Immunoglobulin E
N-ERD: NSAID-Exacerbated Respiratory Disease
CRSwNP: Chronic rhinosinusitis with nasal polyps
NSAID: Non-steroid anti-inflammatory drug
OCS: Oral corticosteroid
PROXIMA: Patient-Reported Outcomes and Xolair In the Management of Asthma
SD: Standard deviation
