# Supplementary figures and images for: Effectiveness of omalizumab in patients with severe allergic asthma with and without chronic rhinosinusitis with nasal polyps: a PROXIMA study post hoc analysis
Source: Clin Transl Allergy. 2020 Jun 26;10:25. doi: 10.1186/s13601-020-00330-1 (PMC7318524; doi:10.1186/s13601-020-00330-1)

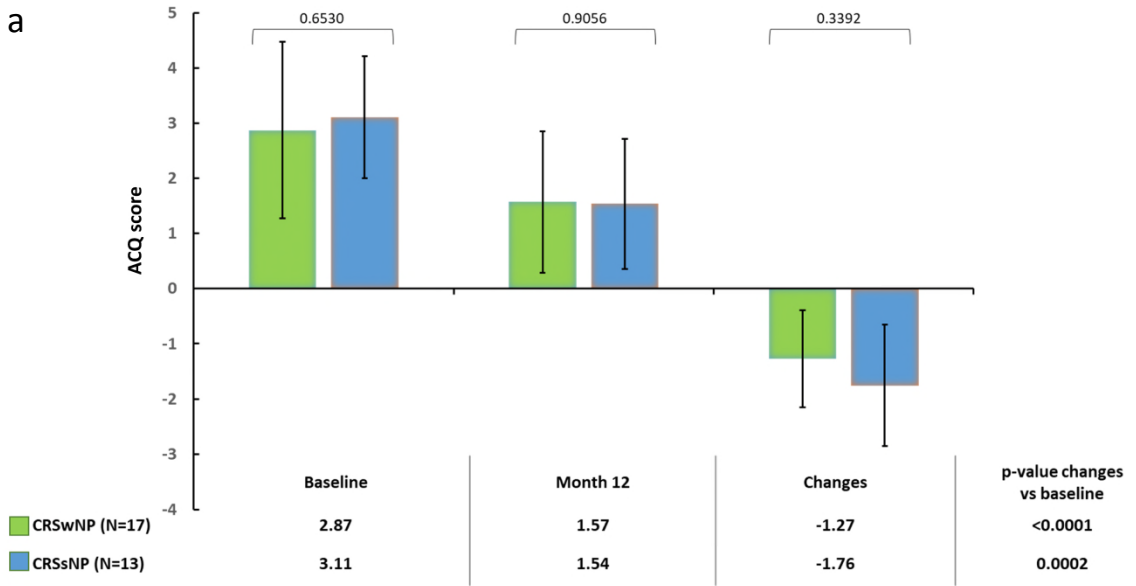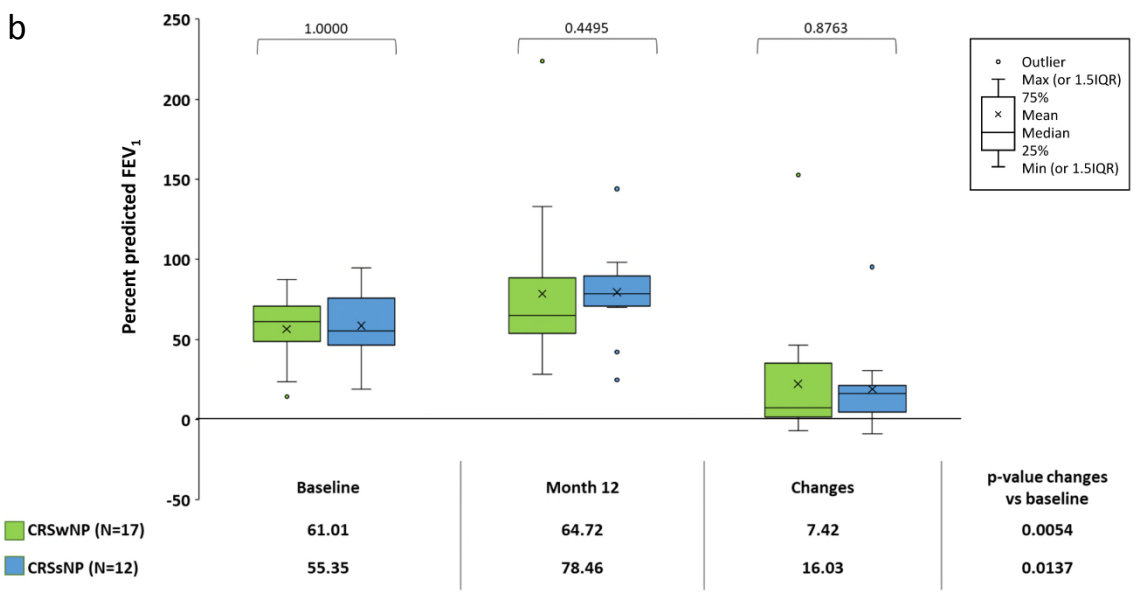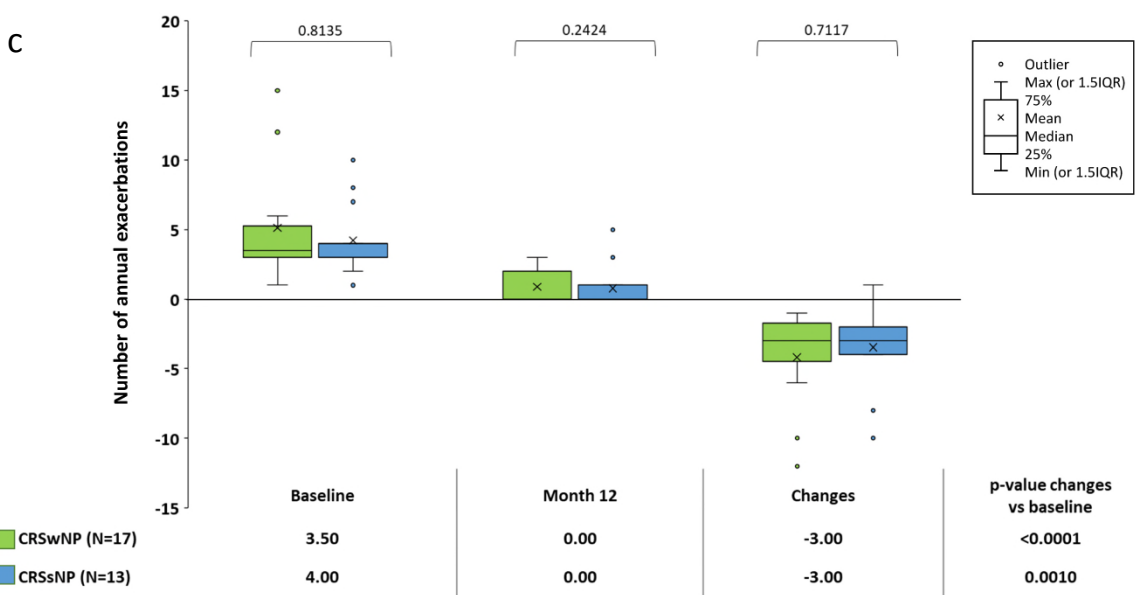

Supplement: Supplementary file 1 — Additional file 1: Figure S1. Efficacy comparison of outcome parameters at baseline and 12 months after omalizumab treatment in patients with chronic rhinosinusitis with nasal polyps (CRSwNP) compared with chronic sinusitis/rhinosinusutis without (CRSsNP). (A) Mean Asthma Control Questionnaire scores, and the change from baseline in ACQ score; (B) Median percent predicted forced expiratory volume in 1 s (FEV1), and change from baseline in percent predicted FEV1; (C) Median number of annual exacerbations in the year prior to initiating omalizumab treatment (baseline) and during 12 months’ treatment with omalizumab, and change from baseline. The p-values within cohorts were calculated using a signed rank test and p-values for comparisons between cohorts were calculated using an ANCOVA model on ranks. [file 13601_2020_330_MOESM1_ESM.pdf]

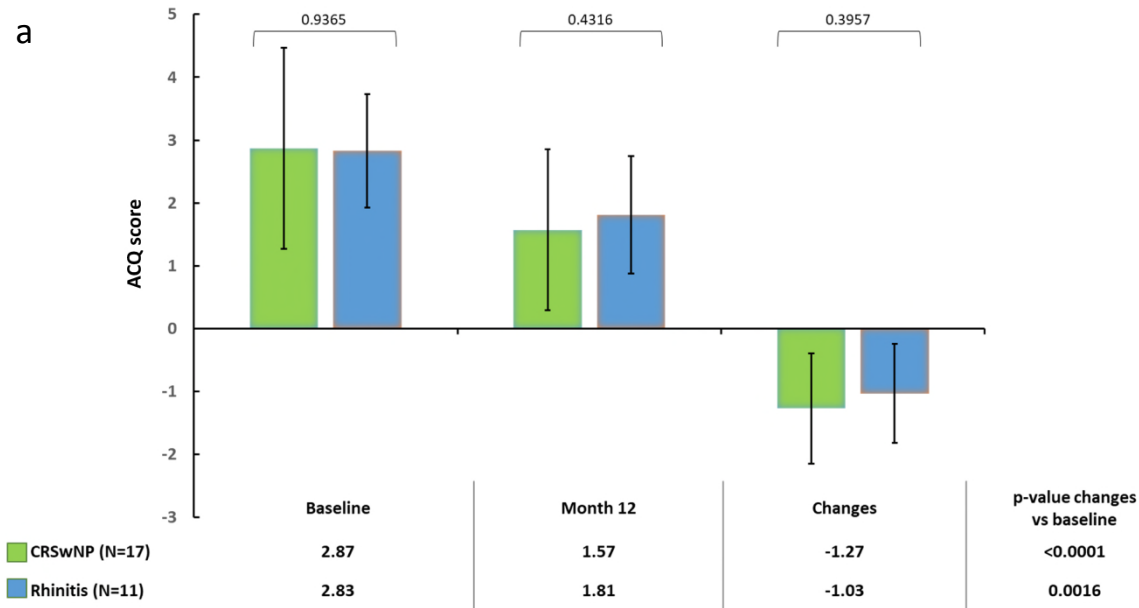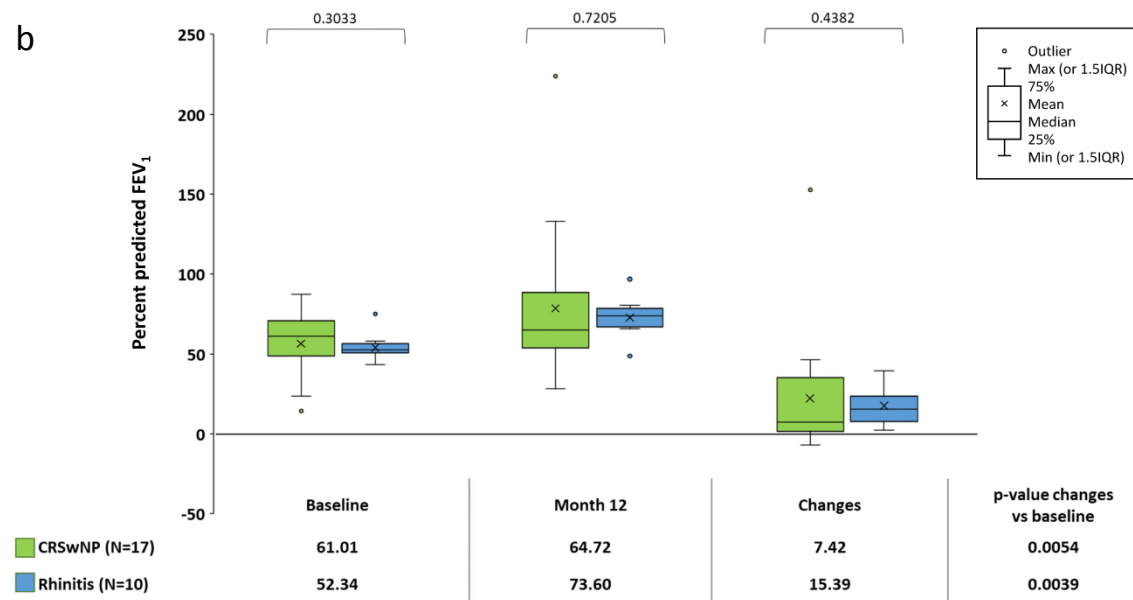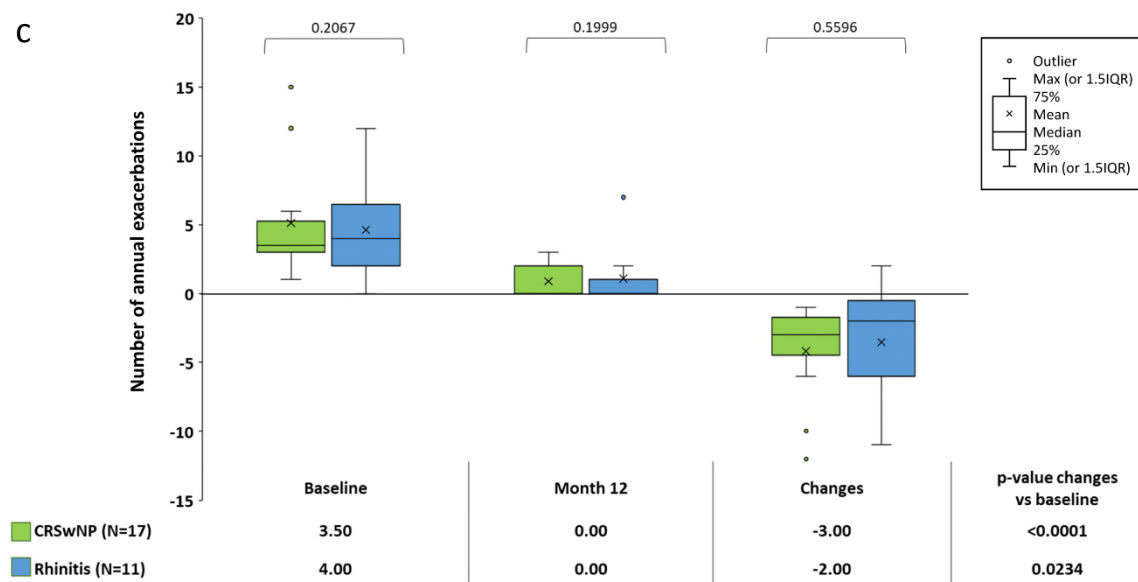

Supplement: Supplementary file 2 — Additional file 2: Figure S2. Efficacy comparison of outcome parameters at baseline and 12 months after omalizumab treatment in patients with chronic rhinosinusitis with nasal polyps (CRSwNP) compared with rhinitis. (A) Mean Asthma Control Questionnaire scores, and the change from baseline in ACQ score; (B) Median percent predicted forced expiratory volume in 1 s (FEV1), and change from baseline in percent predicted FEV1; (C) Median number of annual exacerbations in the year prior to initiating omalizumab treatment (baseline) and during 12 months’ treatment with omalizumab, and change from baseline. The p-values within cohorts were calculated using a signed rank test and p-values for comparisons between cohorts were calculated using an ANCOVA model on ranks. [file 13601_2020_330_MOESM2_ESM.pdf]

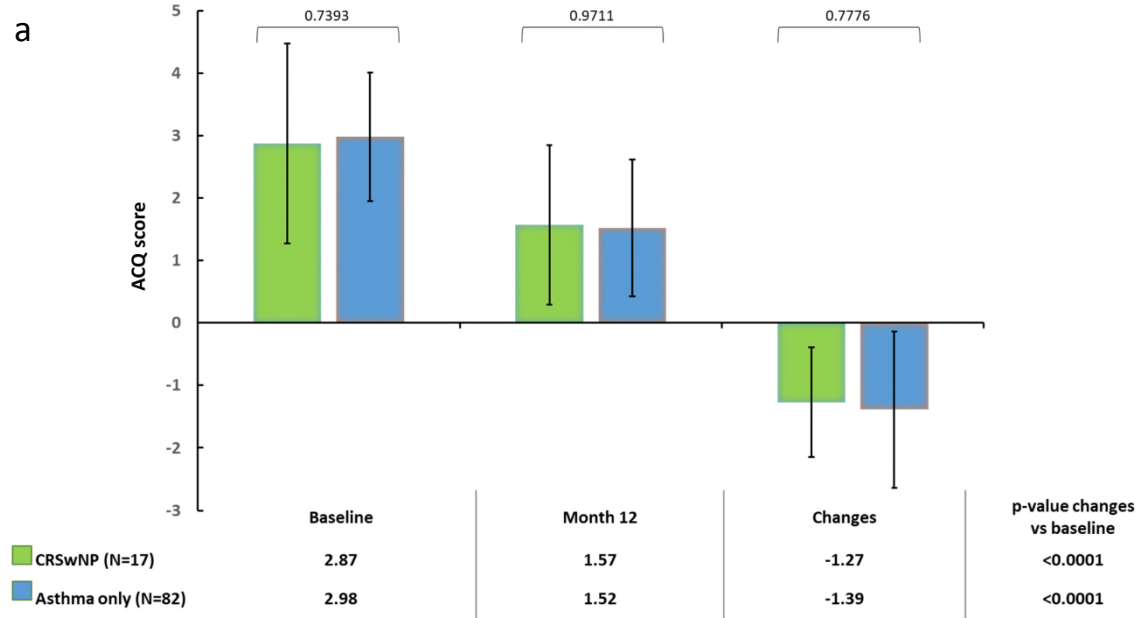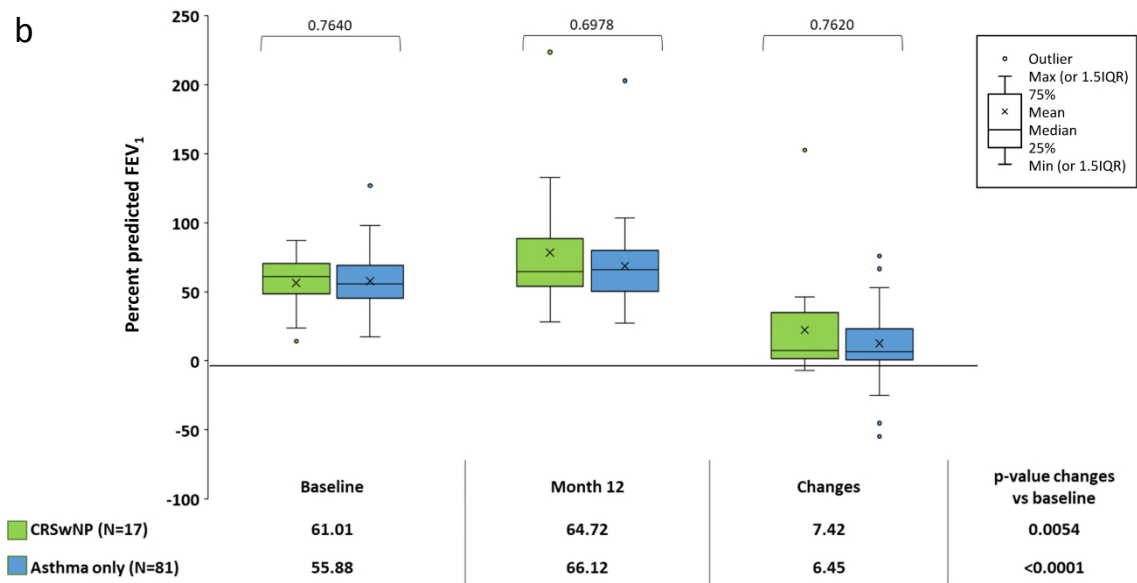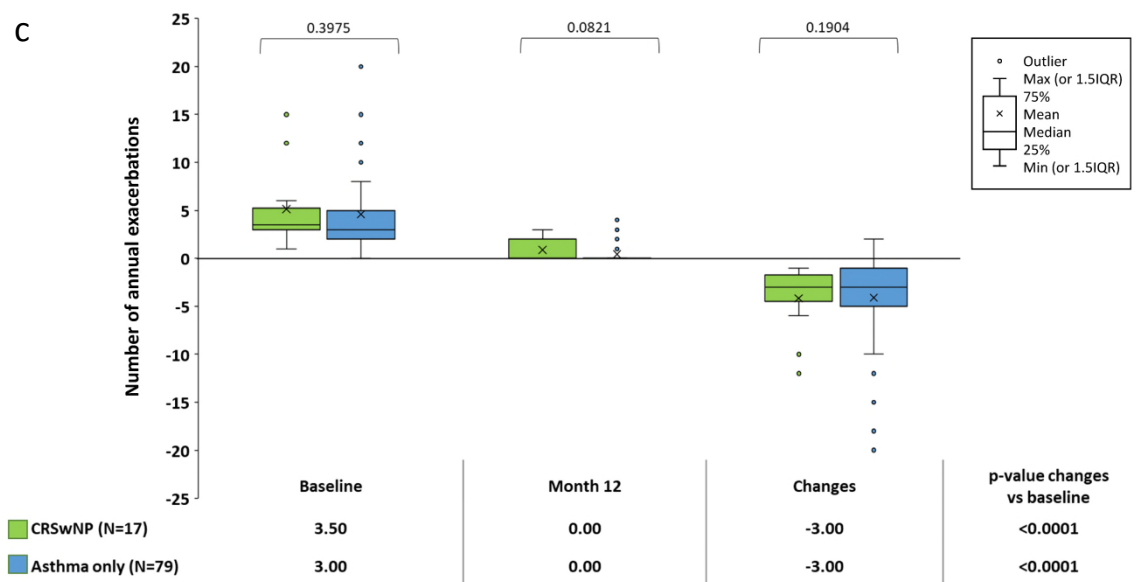

Supplement: Supplementary file 3 — Additional file 3: Figure S3. Efficacy comparison of outcome parameters at baseline and 12 months after omalizumab treatment in patients with chronic rhinosinusitis with nasal polyps (CRSwNP) compared with severe asthma (no chronic rhinosinusitis or nasal polyps). (A) Mean Asthma Control Questionnaire scores, and the change from baseline in ACQ score; (B) Median percent predicted forced expiratory volume in 1 s (FEV1), and change from baseline in percent predicted FEV1; (C) Median number of annual exacerbations in the year prior to initiating omalizumab treatment (baseline) and during 12 months’ treatment with omalizumab, and change from baseline. The p-values within cohorts were calculated using a signed rank test and p-values for comparisons between cohorts were calculated using an ANCOVA model on ranks. [file 13601_2020_330_MOESM3_ESM.pdf]
